# A systematic review and meta-analysis on the prevalence of dietary supplement use by military personnel

**DOI:** 10.1186/1472-6882-14-143

**Published:** 2014-05-02

**Authors:** Joseph J Knapik, Ryan A Steelman, Sally S Hoedebecke, Emily K Farina, Krista G Austin, Harris R Lieberman

**Affiliations:** 1 US Army Research Institute of Environmental Medicine, Natick, MA, USA; 2 US Army Institute of Public Health, Aberdeen Proving Ground, MD, USA; 3 Serenity Hill Nutrition, Street, MD, USA; 4 Oak Ridge Institute for Science and Education, Belcamp, MD, USA; 5 Research Physiologist, ORISE Knowledge Preservation Fellow, USARIEM, 42 Kansas Street, Natick, MA, USA

**Keywords:** Vitamins, Minerals, Multivitamins, Vitamin C, Vitamin E, Calcium, Iron, Protein, Creatine, Sport drink

## Abstract

**Background:**

Although a number of studies have been conducted on the prevalence of dietary supplement (DS) use in military personnel, these investigations have not been previously summarized. This article provides a systematic literature review of this topic.

**Methods:**

Literature databases, reference lists, and other sources were searched to find studies that quantitatively examined the prevalence of DS use in uniformed military groups. Prevalence data were summarized by gender and military service. Where there were at least two investigations, meta-analysis was performed using a random model and homogeneity of the prevalence values was assessed.

**Results:**

The prevalence of any DS use for Army, Navy, Air Force, and Marine Corps men was 55%, 60%, 60%, and 61%, respectively; for women corresponding values were 65%, 71%, 76%, and 71%, respectively. Prevalence of multivitamin and/or multimineral (MVM) use for Army, Navy, Air Force, and Marine Corps men was 32%, 46%, 47%, and 41%, respectively; for women corresponding values were 40%, 55%, 63%, and 53%, respectively. Use prevalence of any individual vitamin or mineral supplement for Army, Navy, Air Force, and Marine Corps men was 18%, 27%, 25%, and 24%, respectively; for women corresponding values were 29%, 36%, 40%, and 33%, respectively. Men in elite military groups (Navy Special Operations, Army Rangers, and Army Special Forces) had a use prevalence of 76% for any DS and 37% for MVM, although individual studies were not homogenous. Among Army men, Army women, and elite military men, use prevalence of Vitamin C was 15% for all three groups; for Vitamin E, use prevalence was 8%, 7%, and 9%, respectively; for sport drinks, use prevalence was 22%, 25% and 39%, respectively. Use prevalence of herbal supplements was generally low compared to vitamins, minerals, and sport drinks, ≤5% in most investigations.

**Conclusions:**

Compared to men, military women had a higher use prevalence of any DS and MVM. Army men and women tended to use DSs and MVM less than other service members. Elite military men appeared to use DSs and sport drinks more than other service members.

## Background

Dietary supplements (DSs) are commercially available products that are consumed as an addition to the usual diet. DSs include ingredients such as vitamins, minerals, herbs (botanicals), amino acids, and a variety of other substances [1]. Marketing claims made for various DSs include the ability to improve overall health status, enhance cognitive or physical performance, increase energy, promote loss of excess weight, attenuate pain, and a variety of other favorable outcomes. The Dietary Supplement Health and Education Act of 1994 [2] established the regulatory framework for DSs in the United States (US). Since this act became law, US sales of DSs have increased from $4 billion in 1994 to $30 billion in 2011 [3,4], an approximate 8-fold increase over 17 years.

Patterns of DS use may differ among distinctive subpopulations. Like athletes, military personnel often have occupational tasks that require intense and prolonged periods of physical activity. Like athletes, service members may use DSs that have purported ergogenic effects to enhance their occupational performance [5-9]. Unlike athletes, service members may be working in austere and hostile surroundings under extreme environmental conditions with high risk of injury. As a result, military personnel may use DSs that purportedly enhance health or performance under these conditions. In contrast, the general US population appears to consume DSs primarily for health reasons with only minor concern for performance enhancement [10,11].

This paper presents a systematic literature review describing the prevalence of DS use in military personnel. No systematic review on this topic has previously been performed. Data collected by our group suggests that the use of DSs by military personnel may exceed that of civilian populations, and that selected subgroups within the military may have even higher DS use than the general military population [8,12].

## Methods

Literature searches were conducted in PubMed, Ovid MEDLINE (including OLDMEDLINE), OVID Healthstar, PsycINFO, Cumulative Index to Nursing and Allied Health Literature (CINAHL), the Defense Technical Information Center (DTIC), and publications from the National Institute of Medicine. No limitations were placed on the dates of the searches with the final search completed in January 2014. To assure that descriptors were all inclusive, we examined Medical Subject Headings for “military personnel” and “DSs” in PubMed. Largely as a result of this examination, keywords selected for the search included military personnel, soldier, sailor, airmen, marine, armed forces personnel, coast guard, submariners, Navy, and Air Force personnel combined with nutrition, DS, supplement, vitamin, mineral, amino acid, protein, herb, herbal, sport drink, sport bar, nutriceuticals, neutraceuticals, food supplements, and food supplementation. To find additional studies, the reference lists of the articles obtained were searched as was the literature database of an investigator with extensive experience with DSs. In several cases, authors were contacted to obtain information that was not included in the article.

Articles were selected for the review if they were:

• Written in English,

• Provided a quantitative assessment of the prevalence of DS use or prevalence could be calculated from data in the article, and

• Participants were military personnel.

Studies were exclude if:

• Participants were other than military personnel,

• The study that did not allow separation of military personnel from others in the study,

• Prevalence could not be calculated as a percent of the total sample in the study, or

• The study that did not include specific DSs.

Data in which DSs were described by terms like “antioxidant”, “pro-performance”, “herbal supplement”, “ergogenics”, “thermogenics”, “bodybuilding”, and the like, were not included in this review because the type of DS was not specific. Exceptions were general categories of vitamins, minerals, sport drinks, sport bars, and energy drinks which were included because so many studies reported these.

Titles were first examined and abstracts were reviewed if the article appeared to involve military personnel and either nutrition or DSs. The full text of the article was retrieved if there was a possibility that DSs were included within the investigation. Quantitative prevalence data were obtained from the text of the article, from tables, or from graphs. If the data was in graphic form, prevalence was estimated from the vertical axis of the graph. Where multiple publications were found on a single study, all individual DS prevalences reported in any of the reports were included in the data extraction. The prevalence of a single type of DS reported in multiple publications from a single study was considered only once in the data extraction and analysis.

The methodological quality of the investigations was assessed using the technique of Loney et al. [13], which was developed specifically for rating prevalence investigations. Studies were graded on an 8-point scale which included assessments for sampling methods, sampling frame, sample size, measurement tools, measurement bias, response rate, statistical presentation, and description of subject sample. The 8 items were rated as either “yes” (1 point) or “no” (no point), based on specific criteria. Thus, the maximum possible score was 8. Three authors independently rated each of the selected articles. Following the independent evaluations, the reviewers met to examine the scores and to reconcile major differences. The average score of the three reviewers served as the methodological quality score. Scores were converted to a percent by dividing the average score for each study by 8 and multiplying by 100%.

The summary statistic derived from each study was the prevalence of specific DS use. This was calculated as the ∑ of individuals using the supplement/∑ of the entire sample × 100%. This expressed use prevalence as a percent of the sample. Data in some studies required recalculation because authors expressed the data as a percent of DS users rather than as a percent of the total sample. Tables were constructed, one containing methodology of each study, and the other containing the prevalences of the DSs reported in the studies. In the methodological table, the “response rate” was calculated as the subjects whose data were used in the investigation divided by the total number of subjects who were asked to participate. The response rates reported by some authors had to be recalculated because the authors reported the number of responses received without considering data that was discarded (e.g., incomplete or improperly completed questionnaires). The prevalence table included columns for the most commonly reported DSs in all articles. These included “any” DS, any vitamins or minerals, multivitamins/multiminerals (MVM), specific vitamins and minerals, creatine, proteins, amino acids, and specific herbal supplements. In cases where a specific supplement was not discussed in a study, the space in the prevalence table was left blank. In studies where at least 4% of the sample used a particular DS not contained in the table columns, that DS was listed in the last column of the prevalence table. Where possible, studies were separated by sex and specific military subgroups (e.g., Army, Navy, Air Force, Marine Corps, Army Rangers, Army Special Forces, Navy Special Operations). In a number of cases the study authors had not separated the data into these categories and so the data was presented as combined. Data were compiled by year to examine if any temporal trends could be discerned.

The Comprehensive Meta-Analysis Statistical Package, Version 2 (Biostat, Englewood NJ). was used to perform meta-analysis on specific groups and specific DSs where there were at least two studies and where subjects were asked about “current” DS use or use ≥1 time/week. A random model was used that involved providing the number of service members using the DS and those not using the DS to produce a summary prevalence estimate (SPE) with a summary 95% confidence interval (S95% CI) that represented the pooled results from the individual investigations. Homogeneity of the prevalence estimates from the studies was assessed using the Q statistic. To examine possible temporal trends, the prevalence of DSs reported in at least 3 studies were plotted by publication year. Curve fitting procedures were applied to these plots including linear, logarithmic, and second order polynomial fits.

## Results

**Figure 1:**
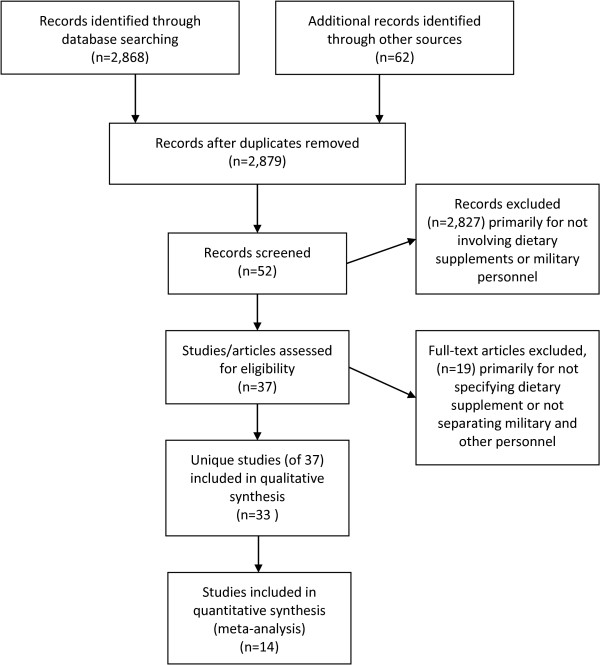
Records included and excluded at each stage of literature review.

The search produced 2,930 potential publications. Figure 1 shows the number of publications included and excluded at each stage of the literature search. Thirty-three unique investigations in 38 publications met the review criteria. Seven reports were in government technical reports [14-20], two were only in abstract form [21,22], 9 were in an Institute of Medicine report on the use of DSs in military personnel [23-31], and 20 reports were in peer-reviewed journal articles [5-9,12,32-45]. Three individual studies had two reports each [15,19,25,30,32,38] and one produced three relevant publications [8,41,42].

Two studies that involved DS use in military groups were excluded. One study of repatriated Vietnam prisoners-of- war was not included because of the unusual circumstances and because the vitamin intake was not voluntary in some cases [46]. Also excluded was one study that asked physicians about DSs reported by patients, as opposed to participants self-reporting their DS use [47].

Table 1 shows the subjects and methods used to obtain data from the 33 unique investigations. Most studies involved US service members, but two studies were conducted among deployed British soldiers [7,40] and one in Macedonian Special Operations Soldiers [45]. Among the US service members, several studies examined elite military service members including Army Rangers [5,22,27,30,38], Army Special Forces [12,28], and Navy Sea, Air, Land (SEAL) personnel [36]. Other studies involved general samples of Army soldiers [6,8,14,16,23,26], Air Force personnel [17,31], Navy [37] and Marine Corps personnel [9,18,37], Marine and Air Force trainees [20,36,39], senior military officers [29], military officers in training [15,35], men initiating training to become Ranger and Special Forces soldiers [33], and multiple service groups [19,24,25,34,43,44].

**Table 1:** Methods in investigations of DS use by military personnel

**Study**	**Subjects**	**Methods for collecting supplement information**	**Reporting timeframe**	**Response rate (%) - proportion of population participating in investigation**	**Methodological quality score (%)**
Carlson et al. [14]	43 ♂ US Army senior non-commissioned officers	Questionnaire with items on DS use	Current Use	86	75
Klicka et al. [15]	119 ♂ & 86 ♀ cadets at the US Military Academy at West Point NY	7-day dietary record with interview	Use over 7 days	Not provided	38
Schneider et al. [21]	91 ♂ US Navy SEAL personnel	Questionnaire focused on DS use	Current Use	Not provided	25
Kennedy and Arsenault [33]	2,215 ♂ US Army soldiers entering Special Forces and Ranger training	Questionnaire focused on DS use	Current use, ≥ 1 time/wk, within last 3 months	99	66
Warber et al. [16]	2,547 ♂ & 494 ♀ US Army soldiers from 32 Army installations world-wide	Questionnaires with items on DSs	Current use ≥1 time/wk	Not provided	59
McGraw et al. [22]	367 ♂ US Army Rangers	Questionnaire focused on DS use	Use in past 6 months	Not provided	41
Sheppard et al. [34]	102 ♂ & 31 ♀ US service members using health clubs	Questionnaire focused on creatine and use of other DSs	Current use	70	66
Arsenault & Cline [35]	50 ♀ officers in Basic Officer Training	7-day dietary record with interview	Use over 7 day period	Not provided	42
Stevens and Olsen [36]	439 ♂ & 60 ♀ US marine or AF basic trainees	Questionnaire focused on ergogenic DS use	Any use in lifetime	91	91
Shanks [17]	224 ♀ active duty US AF women	Questionnaire focused on herbal therapy	Use in the last 2 years	45	75
Bovill et al. [12]	119 ♂ US Army Special Forces soldiers	Questionnaire on nutrition and DS use	Current use	Not provided	29
Deuster et al. [5]	38 ♂ US Army Rangers at Fort Bragg, North Carolina	General health questionnaire with items on DSs	Daily Use	Not provided	29
Brasfield [6]	750 ♂ & 124 ♀ US Army soldiers from 16 Army posts	Questionnaire focused on DS use	Current use ≥1 time/wk	58	59
Castillo et al. [18]	1,326 ♂ & 120 ♀ US marines at Camp Pendleton, California	Questionnaire (Marine Health Behavior Survey) with items on DSs	Use in last year	85	59
Bray et al. [19,25]	12,119 ♂ & 4,027 ♀ US quad-service members (23% Army; 29% Navy, 21% Marine Corps, 28% AF)	Questionnaire with items on DS use	Current use ≥1 time/wk in last year	53	88
Smith et al. [37]	1,009 ♂ and 296 ♀ US Navy and Marine Corps personnel (72% Navy, 22% Marine Corps, 6% Navy Reserve/Guard)	Questionnaire with items on DS use	Use in past year	35	79
Johnson et al. [30,38]	294 ♂ US Army Rangers	Questionnaire focused on DS use	Current Use	39	41
Corum [23]	3,789 ♂ & 1,146 ♀ US Army soldiers assigned in Europe	Questionnaire focused on DS use	Current use	Not provided	38
French [24]	376 US service members, active duty, National Guard, or reserves	On-line questionnaire focused on DS use	Use in past 3 months	60	38
Lieberman [29]	284 ♂ & 31 ♀ US senior military officers at the US Army War College, all services	Questionnaire focused on DS use	Use ≥ 1 time/wk	Not provided	34
Lieberman [27]	768 ♂ US Army Rangers	Questionnaire focused on DS use	Use ≥1 time/wk	Not provided	25
Lieberman [28]	152 ♂ US Army Special Forces soldiers	Questionnaire focused on DS use	Use ≥1 time/wk	Not provided	29
Lieberman [26]	444 ♂ & 40 ♀ US Army soldiers	Questionnaire focused on DS use	Use ≥1 time/wk	80	46
Thomasos 2008 [31]	10,985 US AF personnel at 27 major installations	Questionnaire focused on DS use	Current use	Not provided	38
Young and Stephens [39]	236 ♂ & 83 ♀ US Marine Corps recruits entering basic training	Questionnaire focused on DS use	Use at any time in past	65	54
Boos et al. [7]	889 ♂ & 128 ♀ UK Soldiers deployed in Basra, Iraq	Questionnaire focused on DS use	Current Use	66	66
Lieberman et al. [8,41,42]	859 ♂ &131 ♀ US Army soldiers from 11 locations including two overseas; 17 ♂ Special Forces soldiers	Questionnaire focused on DS use	Use ≥1 time/wk in last 6 months	~80	71
Wells & Webb [20]	197 US AF trainees in Tactical Air Control Party training	Questionnaire with items on DSs	Current Use	Not provided	41
Boos et al. [40]	78 ♂ & 9 ♀ UK soldiers deployed in Afghanistan	Questionnaire focused on DS use	Current use	58	29
Jacobson et al. [43]	72,718 ♂ & 33,980 ♀ US service members (active & reserve) enrolled in the Millennium Cohort Study	Questionnaire including item on DS use	Use in last 12 months	68	88
Toblin et al. [44]	988 ♂ US Army soldiers and marines deployed to Afghanistan	Questionnaire including item on energy drinks	Daily Use	79	71
Kjertakov et al. 2013 [45]	132 ♂ Macedonian Special Operations soldiers	Questionnaire focused on DS use	Use in last 3 months	Not provided	41
Cassler et al. [9]	310 ♂ & 19 ♀ US marines deployed in Afghanistan (Camp Leatherneck)	Questionnaire focused on DS use	Use in last 30 days	Not provided	38

*Abbreviations*: ♂ = men, ♀ = women; DS(s) = dietary supplement(s); US = United States; UK = United Kingdom; AF = Air Force; SEAL = Sea, Air, Land (US Navy special operations personnel); wk = week; NY = New York.

In most unique studies (n = 31), the data were collected by having service members self-report their DS use on questionnaires. Most of the questionnaires were specifically designed to obtain information on DSs and focused on this topic [6-9,12,17,21-24,26-31,33,34,36],[38-42,45]. Other studies obtained DS information from questionnaires that had items on DS use but were designed for more general purposes, often to collect a range of nutritional measures [5,14,16,18-20,25,37,43,44]. Two unique investigations obtained DS information from 7-day food records complimented with interviews by dietitians [15,32,35].

The reporting timeframe differed among studies. In many investigations, service members reported DS use ≥1 time/week, or these data could be calculated from the information provided in the articles [6,8,16,19,25-29,33,41,42]. Other studies examined current use but did not clearly define the frequency of use [7,12,14,23,24,30,31,34],[38,40]. In other studies, service members reported use in the last 7 days [15,32,35], last month [9], last 3 months [24,45], last 6 months [22], last 12 months [18,37,43], or last two years [17]. Two studies examined the lifetime prevalence of DS use in Marine and Air Force basic trainees [36,39]. Two studies reported daily use [5,44] and in one study the reporting timeframe was not stated [21].

The response rate was not provided in 15 unique studies [5,9,12,15,16,20-23,27-29,31],[35,45]. In the 18 other unique investigations, the response rates ranged from 35% [37] to 99% [33] with only 10 unique investigations having response rates ≥66% [7,8,14,18,26,33,34,36],[43,44].

Rating from the methodological quality reviews ranged from 25% to 91% of available points, with an average ± standard deviation rating of 52 ± 20%. Only 8 studies (24%) had a rating of 70% or better. Several studies were only reported in an Institute of Medicine publication on DS use in the military [48] and received relatively lower scores because of the lack of detail provided [23,24,26-29,31]. The study by Bray et al. [19] was particularly well conducted in that the investigators attempted to collect a random sample of the entire military and clearly outlined their sampling methods, sampling frame, questionnaire, and results.

Table 2 shows available data on the prevalence of DS use by service members. Twenty-six unique investigations provided the prevalence of “any” DS use [5-9,12,15,18-22,24,26-29,31,33],[35,36,38-40,43,45] and 17 reported on multivitamin use [6-9,12,16,19,21-24,26-29,33,45]. Only 10 unique investigations reported on specific vitamins and mineral supplements [6,12,22-24,26,28,29,33,45] and 7 reported on specific herbal supplements [6,17,23,29,33,39,45]. Nineteen unique investigations reported on supplementation with creatine [5-7,12,18,21-24,26-28,33,34,36],[38-40,45] and 20 reported on amino acid and/or protein supplements [5-9,12,16,21-24,26-28,33,34,38-40],[45]. Finally, supplementation with sport drinks were reported in 11 investigations [5,8,12,21,23,26-29,39,45], sport bars in 10 studies [8,12,16,21-23,26-28,33], and energy drinks in 3 investigations [8,9,44]. None of the studies identified the use prevalence of specific brands of MVM, vitamins, minerals, amino acids, proteins, botanicals, sport drinks or sport bars.

**Table 2:** Prevalence of dietary supplement use by military personnel

**Study**	**Subjects**	**Proportion of entire sample reporting use (%)**																	
		**Any DS Use**	**Any Vit (V), Minl (M), or Vit/Minl (VM) Suppl**	**MV or MVM Suppl**	**Vit A**	**Vit B or B Cmpx**	**Vit C**	**Vit D**	**Vit E**	**Fe**	**Ca**	**Zn**	**Creatine**	**Amino acids (A), Prot (P), or Amino acids & Prot (AP)**	**Ginkgo Biloba**	**Ginseng**	**Sport drink**	**Sport bar**	**Other DSs in study**
Carlson et al. [14]	43 ♂ US Army senior non-commissioned officers		VM-7																
Klicka et al. [15,32]	119 ♂ cadets at USMA	14																	
	86 ♀ cadets at USMA	35																	
Schneider et al. [21]	91 ♂ SEAL personnel	78		26									32	AP-12			19	40	
Kennedy & Arsenault [33]	2,215 ♂ US Army Special Forces and Rangers in training	64		30	5^b^	3	13		6	3	5	3	15	A-9^a^		5		11	Cr-4
Warber et al. [16]	2,547 ♂ US Army Soldiers		V-18	24										A-10				8	
	494 ♀ US Army Soldiers		V-24	35										A-4				3	
McGraw et al. [22]	367 ♂ US Army Rangers	36		16			7						19	AP-14				6	
Sheppard et al. [34]	102 ♂ & 31 ♀ US military health clubs users		V-65 M-47										29	P-45					Caf-32
Arsenault & Cline [35]	50 ♀ US officers in Basic Officer Training	38																	
Stevens & Olsen [36]	439 ♂ & 60 ♀ US Marine Corps or AF basic trainees	41											23						AD-8
Shanks [17]	224 ♀ US active duty AF women														5	5			Eph-5; Gar-4; Ech-5; SJW-5
Bovill et al. [12]	119 ♂ US Army Special Forces soldiers	90		55			20		12		9		18	P-24			71	52	
Deuster et al. [5]	38 ♂ US Army Rangers	82											13	P-24			82		Eph-13
Brasfield [6]	750 ♂ US Army soldiers	59		33	8	7^c^	17		10	7	12	3	16	A-6	6	14			K-7; Vit B_6_-6; CP-4; Eph-13; Gar-7; Ech-4
	124 ♀ US Army soldiers	70		40	7		15		7	12	14					6			
Castillo et al. [18]	1,326 ♂ & 120 ♀ US marines	54	VM-13										18						Eph-24; AD-5
Bray et al. [19]	12,119 ♂ US quad-service members	58	VM-25	43															
	4,027 ♀ US quad-service members	71	VM-37	56															
	2,818 ♂ Army Soldiers	55	VM-24	38															
	821 ♀ Army Soldiers	66	VM-34	50															
	3,341 ♂ Navy Sailors	60	VM-27	46															
	1,286 ♀ Navy Sailors	71	VM-36	55															
	2,767 ♂ Marines	61	VM-24	41															
	589 ♀ Marines	71	VM-33	53															
	3,193 ♂ AF Airmen	60	VM-25	47															
	1,331 ♀ AF Airmen	76	VM-40	63															
Smith et al. [37]	1,009 ♂ and 296 ♀ US Navy and Marine Corps personnel		V-12																
Johnson et al. [30,38]	294 ♂ US Army Rangers	56											26	P-35 A-4					
Corum [23]	3,789 ♂ & 1,146 ♀ US Army Soldiers assigned in Europe			34	13		24			14	19		13	P-14	4	7	43	17	K-12; Ech-4; Gar-5; Vit B_6_-12; Caf-18
French [24]	376 US service members	69		57		8		3	9		13		6	P-14 A-4					Ω3FA-9; GlCon-7; FSO-4
Lieberman [29]	284 ♂ US senior military officers	71		39	5	6	17		22		5				5		10		Gar-6
	31 ♀ US senior military officers	81		52	16	36^d^	29		32		32								Mg-13
Lieberman [27]	768 ♂ US Army Rangers	81		23									19	PA-18			41	6	AD-7
Lieberman [28]	152 ♂ US Army Special Forces Soldiers	65		32			11		7				16	P-16			36	15	AD-6
Lieberman [26]	444♂ US Army Soldiers	55		30	5		13	5	6		6		5	P-13			20	5	
	40 ♀ US Army Soldiers	70		28		23^e^	13	8	8	10	15			P-8			28		
Thomasos [31]	10,985 US AFpersonnel	69																	
Young & Stephens [39]	236 ♂ & 83 ♀ US Marine Corps recruits entering basic training	50	V-26									3	26	P-43	6		36		NO-16;Glu-16; GlCon-9
Boos et al. [7]	889 ♂ & 128 ♀ UK Soldiers deployed in Iraq	32		2									13	P-19 A-18					Caf-4
Lieberman et al. [8,41,42]	859 ♂ US Army Soldiers	53^f^	VM-17	37										PA-20			23	6	ED-41
	131 ♀ US Army Soldiers	57^f^	VM-23	41										PA-9			24	4	ED-25
	17 ♂ US Special Forces Soldiers	77 ^f^	VM-64	64										PA-47			32	7	
Wells & Webb [20]	197 US AF trainees in Tactical Air Control Party training	73																	
Boos et al. [40]	78 ♂ & 9 ♀ UK Soldiers deployed in Afghanistan	40											14	PA-34					CP-15
Jacobson et al. [43]	72,718 ♂ and 33,980 ♀ US service members (active & reserve)	47																	
Toblin et al. [44]	988 ♂ deployed US Army soldiers and marines																		ED-45
Kjertakov et al. [45]	78 ♂ Macedonian Rangers	64		47	13	14	44		9	8	18	3	3	P-8A-5		0	15		VitB_6_-14; Vit B_12_-6; Mg-10
	54 ♂ Macedonian Special Forces Soldiers	70		54	13	24	54		15	13	11	2	2	P-7 A-11^a^		2	15		VitB_6_-13; Vit B_12_-9; Mg-16; Se-4; Glu-4
Cassler et al. [9]	310 ♂ US marines deployed in Afghanistan	72		47										P-64					ED-42
	19 ♀ US marines deployed in Afghanistan	42																	

*Abbreviations*: ♂ = men, ♀ = women; DS = dietary supplement; Vit or V = vitamin; Minl or M = mineral; VM = vitamin and/or mineral; MV = multivitamin; MVM = multivitamin/multimineral; A = amino acid; Prot or P = protein; AP = amino acids and protein; cmpx = complex; Fe = iron; Ca = calcium; Zn = zinc; Mg = magnesium; Cr = chromium; Se = selenium; K = potassium; Caf = caffeine; ED = energy drink; Gar = garlic; AD = androstenedione; Eph = ephedrine; FSO = flax seed oil; Glu = glutamine; Ech-echinacea; SJW = Saint John’s Wort; Ω3FA = omega-3-fatty acid; GlCon = glucosamine chondroitin; NO = nitric acid; CP = chromium picolinate; AF = Air Force; SEAL = Sea, Air, Land (US Navy special operations personnel); UK = United Kingdom; USMA = United State Military Academy.

Notes: ^a^Combines amino acids and branched chain amino acids; ^b^Combines β-carotene and Vit A; ^c^Combines B-complex, folic acid, and pantothenic acid; ^d^Combines folate, B-complex, and Vit B_6_; ^e^Combines folate and Vit B_6_; ^f^Excludes sport drinks/bars/gels.

Table 3 shows summary data on US military DS use where questionnaires had asked about “current use” or use ≥1 time per week. Only 13 studies were included in these meta-analyses (Figure 1) because the other studies used different reporting timeframes or reported on a DS that was not included in any other study. Additional data from Bray et al. [19] was included in the table because this investigation was the only one to ask about use of any DS, multivitamins, or any vitamin or mineral supplement ≥1 time/week among Navy, Air Force, and Marine personnel. Meta-analyses indicated that the prevalence of any DS use was relatively consistent among Army studies. Among men in the four military services, use of supplements of any kind ranged from 53% to 61% of the surveyed groups; among women, use of supplements of any kind ranged from 66% to 76%. Female Army officers in training tended to have a much lower use of DSs. Reported supplement use among elite Army and Navy groups was less homogenous ranging from 56% to 90% of the surveyed groups, but was, in the main, higher than general military samples.

**Table 3:** Summary data on prevalence of dietary supplement use by military personnel by gender and service

**Dietary supplement**	**Group**	**Studies (reference number)**	**Individual study prevalence, mean (95% CI)**	**Total sample size (n)**	**Summary prevalence estimate (95% CI) (%)**	**Homogeneity of summary prevalence estimate**
			**(%)**			**(Q Statistic p-value)**
Any	Army Men	Brasfield [6]	59 (56–63)	4,871	55(53–56)	0.77
		Bray et al. [19]	55 (53–57)			
		Lieberman [26]	55 (50–60)			
		Lieberman et al. [8]	53 (50–56)			
	Navy Men	Bray et al. [19]	60 (58–62)	3,341		
	AF Men	Bray et al. [19]	60 (58–62)	3,193		
	Marine Men	Bray et al. [19]	61 (59–63)	2,767		
	Army Women	Brasfield [6]	70 (62–78)	1,844	65(60–70)	0.14
		Bray et al. [19]	66 (63–69)			
		Lieberman [26]	70 (56–84)			
		Lieberman et al. [8]	57 (49–66)			
	Navy Women	Bray et al. [19]	71 (68–74)	1,286		
	AF Women	Bray et al. [19]	76 (74–78)	1.331		
	Marine Women	Bray et al. [19]	71 (67–75)	589		
	Army Officers in Training, Women	Klicka et al. [15]	35 (25–45)	136	36(28–44)	0.72
		Arsenault et al. [35]	38 (25–51)			
	Navy SEALs	Schneider et al. [21]	78 (70–87)	1,479	76(65–85)	<0.01
	Army SF	Bovill et al. [12]	90 (85–95)			
	Army Rangers	Deuster et al. [5]	82 (70–94)			
	Army Rangers	Johnson et al. [38]	56 (50–62)			
	Army Rangers	Lieberman [27]	81 (78–84)			
	Army SF	Lieberman [28]	65 (57–73)			
	Army SF	Lieberman et al. [8]	77 (57–97)			
Multivitamin	Army Men	Warber et al. [16]	24 (22–26)	7,418	32(26–39)	<0.01
		Brasfield [6]	33 (30–36)			
		Bray et al. [19]	38 (36–40)			
		Lieberman [26]	30 (26–34)			
		Lieberman [8]	37 (34–40)			
	Navy Men	Bray et al. [19]	46 (45–48)	3,341		
	AF Men	Bray et al. [19]	47 (45–49)	3,193		
	Marine Men	Bray et al. [19]	41 (39–43)	2,767		
	Army Women	Warber et al. [16]	35 (31–39)	1,610	40(32–48)	<0.01
		Brasfield [6]	40 (31–49)			
		Bray et al. [19]	50 (47–53)			
		Lieberman [26]	28 (14–42)			
		Lieberman et al. 2010 [8]	41 (33–49)			
	Navy Women	Bray et al. [19]	55 (52–58)	1,286		
	AF Women	Bray et al. [19]	63 (60–66)	1,331		
	Marine Women	Bray et al. [19]	53 (49–57)	589		
	Navy SEALS	Schneider et al. [21]	26 (17–35)	1,147	37(25–52)	<0.01
	Army SF	Bovill et al. [12]	55 (46–64)			
	Army Rangers	Lieberman [27]	23 (20–26)			
	Army SF	Lieberman [28]	32 (25–39)			
		Lieberman et al. [8]	64 (41–87)			
Any vitamin or mineral	Army Men	Carlson et al. [14]	7 (1–13)	3,720	18(13–26)	<0.01
		Bray et al. [19]	24 (22–26)			
		Lieberman et al. [8]	17 (15–20)			
	Navy Men	Bray et al. [19]	27 (25–29)	3,341		
	AF Men	Bray et al. [19]	25 (24–27)	3,193		
	Marine Men	Bray et al. [19]	24 (22–26)	2,767		
	Army Women	Bray et al. [19]	34 (31–37)	952	29(19–41)	0.01
		Lieberman et al. [8]	23 (16–30)			
	Navy Women	Bray et al. [19]	36 (33–39)	1,286		
	AF Women	Bray et al. [19]	40 (37–43)	1,331		
	Marine Women	Bray et al. [19]	33 (29–37)	589		
Vitamin C	Army Men	Brasfield [6]	17 (14–20)	1,609	15(12–20)	0.07
		Lieberman et al. [26]	13 (10–16)			
	Army Women	Brasfield [6]	15 (9–21)	255	15(10–21)	0.19
		Lieberman et al. 2008 [26]	13 (3–23)			
	Army SF	Bovill 2003 [12]	20 (13–27)	271	15(8–26)	0.04
		Lieberman [28]	11 (6–16)			
Vitamin E	Army Men	Brasfield [6]	10 (8–12)	514	8(5–13)	0.12
		Lieberman [26]	6 (4–8)			
	Army Women	Brasfield [6]	7 (3–12)	164	7(4–12)	0.95
		Lieberman [26]	8 (1–16)			
	Army SF	Bovill et al. [12]	12 (6–18)	271	9(6–15)	0.21
		Lieberman [28]	7 (3–11)			
Calcium	Army Men	Brasfield [6]	12 (10–14)	1,194	9(4–17)	<0.01
		Lieberman [26]	6 (4–8)			
	Army Women	Brasfield [6]	14 (8–20)	164	14(10–20)	0.84
		Lieberman[26]	15 (4–26)			
Iron	Army Women	Brasfield [6]	12 (6–18)	164	12(8–18)	0.72
		Lieberman [26]	10 (1–19)			
Protein	Army SF	Bovill et al. [12]	24 (16–32)	271	20(13–30)	0.08
		Lieberman [28]	16 (10–22)			
Protein or amino acid	Navy SEALs	Schneider et al. [21]	18 (15–21)	876	21(11–36)	<0.01
	Army Rangers	Lieberman [27]	12 (5–19)			
	Army SF	Lieberman et al. [8]	47 (23–71)			
Creatine	Army Men	Brasfield [6]	16 (13–18)	1,194	9(3–27)	<0.01
		Lieberman [26]	5 (3–7)			
	Navy SEALs	Schneider et al. [21]	32 (22–42)	1,169	20(15–25)	0.02
	Army SF	Bovill et al. [12]	18 (11–25)			
	Army Rangers	Deuster et al.[5]	13 (2–24)			
	Army Rangers	Lieberman [27]	19 (16–22)			
	Army SF	Lieberman [28]	16 (10–22)			
Sport drinks	Army Men	Lieberman [26]	20 (16–24)	1,303	22(19–25)	0.22
		Lieberman et al. [8]	23 (20–26)			
	Army Women	Lieberman [26]	28 (14–42)	171	25(19–32)	0.24
		Lieberman et al. [8]	24 (17–31)			
	Navy SEALs	Schneider et al. [21]	19 (11–27)	1,147	39(26–55)	<0.01
	Army SF	Bovill et al. [12]	71 (63–79)			
	Army Rangers	Lieberman [27]	41 (38–44)			
	Army SF	Lieberman [28]	36 (28–44)			
	Army SF	Lieberman et al. [8]	32 (10–54)			
Sport bars	Army Men	Warber et al. [16]	8 (7–9)	3,890	7(5–9)	0.03
		Lieberman [26]	5 (3–7)			
		Lieberman et al. [8]	6 (4–8)			
	Army Women	Warber et al. [16]	3 (2–5)	625	3(2–5)	0.65
		Lieberman et al. [8]	4 (1–7)			
	Navy SEALs	Schneider et al. [21]	40 (30–50)	1,147	19(6–46)	<0.01
	Army SF	Bovill et al. [12]	52 (43–61)			
	Army Rangers	Lieberman [27]	6 (4–8)			
	Army SF	Lieberman [28]	15 (9–21)			
	Army SF	Lieberman et al. [8]	7 (1–14)			

*Abbreviations:* AF = Air Force; SEAL = Sea, Air, Land (US Navy special operations personnel); SF = Special Forces; CI = confidence interval.

Table 3 shows prevalence data on MVM use by military personnel. Army studies demonstrated a lack of homogeneity for both men and women. Among men in the four military services, MVM use ranged from 24% to 47% with Army men less likely to consume multivitamins than men in the other services. Among women, MVM use ranged from 28% to 63% in the four services, again with Army women less likely to consume multivitamins than women in other services. Reported MVM use among elite military groups appeared to be similar to that of Army men but the prevalence values lacked homogeneity and ranged from 26% to 64%.

Table 3 shows that studies on the use prevalence of any vitamin or mineral supplement by Army men and women lacked homogeneity. Nonetheless, data suggested women used vitamin and mineral supplements more often than men, regardless of service. Among all four services, prevalence of use of any vitamin or mineral supplement among men ranged from 7% to 27% while for women this range was 23% to 40%.

Table 3 shows other DSs that had multiple studies and fit the table criteria (current use or use ≥ 1 times/week). These included Vitamin C, Vitamin E, calcium, iron, proteins, protein or amino acids, creatine, sport drinks, and sport bars. Multiple studies (i.e., ≥2) were limited to Army men, Army women, and some elite military groups. The prevalence of Vitamin C supplementation was 15% for Army men, Army women, and Special Forces soldiers, although the latter estimate lacked homogeneity. The prevalence of Vitamin E supplementation was similar among Army men and the Special Forces soldiers and slightly higher than that of Army women. Calcium supplementation was slightly more prevalent among Army women compared to Army men, although the two available male studies were not homogeneous. Creatine use appeared more prevalent among samples of elite Army men compared to general male Army samples, although prevalence values differed considerably. When the Navy SEAL study [21] was eliminated from the analysis of creatine prevalence, studies on Rangers and Special Forces soldiers had a SPE = 19% and S95% CI = 16-21 (p = 0.88 for homogeneity). Use prevalence of sport drinks was similar among the general samples of Army men and women. Meta-analysis suggested that elite military group use of sport drinks was higher than in general Army samples but studies of elite groups lacked homogeneity, with individual study prevalences ranging from 19% to 71%. The prevalence of sport bar usage was low among samples of Army men and even lower among Army women. Sport bar usage appeared much higher in some samples of elite military groups but there was a lack of homogeneity with prevalences ranging from 6% to 52%.

The curve fitting procedures did not indicate any significant temporal trends among studies. This can be appreciated by examining the data presented by year in Table 3.

## Discussion

This review demonstrates that the prevalence of DS use was high among members of the military services. Available data indicated that the self-reported prevalence of DS current use or use ≥1 time/week was slightly lower among Army men (55%) than among men in the other 3 military services (~60%). Female service members reported a higher prevalence of DS use than male service members but again, Army women reported a slightly lower use of DSs of any kind (66%) compared to women in the other 3 services (71 to 76%). The pattern of MVM use was similar to that of the overall use prevalence. That is, Army men and women report MVM use prevalences lower than the other services but women in all services report use prevalences higher than their male counterparts in the same service. Prevalence estimates varied in groups of elite service members (Army Rangers, Army Special Forces, and Navy SEALs) but generally, the use prevalence of supplements of any kind was higher (56% to 90%) than that of other service members. Interestingly, male and female Army officers in training tended to have a lower prevalence of DS use, possibly related to the fact that they were eating in military dining facilities and had busy training schedules.

MVM appeared to be the most commonly used DS among the general population of military personnel with 24% to 47% of male and 28% to 63% of female service members using these supplements. This is in consonance with data on athletes [49,50] and the general US population [51-56] where MVM are also the most commonly consumed DS. Table 4 shows summary data from the National Health Interview Surveys (NHIS) [54,55,57] and the National Health and Nutrition Surveys (NHANES) [58-61], both of which are nationally representative samples of the general US population. It was difficult to directly compare military MVM prevalence data to that of these national surveys because the military and civilian studies differed in terms of the reporting timeframe, methods used to collect the data, and the years when the data were collected. Despite these issues, it was possible to make some general observations. The NHANES and NHIS surveys indicated that women were more likely to use DSs than men, in agreement with the military data. Both NHANES and NHIS surveys observed a temporal trend indicating that DS use increased over time, although the most recent NHANES data suggested a leveling off [60,61]. No such temporal trends could be discerned in the military data, possibly because of the shorter time over which the studies were conducted and lack of questionnaire standardization across studies. Use of MVM appeared to be higher in the military compared to these civilian samples.

**Table 4:** Summary data on DS Use in United States national surveys

**Survey**	**Study**	**Survey year(s)**	**N**	**Reporting timeframe**	**Prevalence (%)**									
					**Any VM**		**Multivitamin**		**Vitamin C**		**Vitamin E**		**Calcium**	
					**Men**	**Women**	**Men**	**Women**	**Men**	**Women**	**Men**	**Women**	**Men**	**Women**
National Health Interview Survey (NHIS)	Subar & Block [57]	1987	9,160 ♂, 12,920 ♀	Daily Use	19	27	15	20	7	8	4	5	3	10
	Slesinski et al. [54]	1992	5,120 ♂, 6,885 ♀	Daily Use	20	27	17	22	7	8	4	5	2	8
	Millen et al. [55]	2000	34,085 ♂ & ♀	Daily Use	29	39	24	33	10	12	10	13	4	17
National Health and Nutrition Survey (NHANES)	Koplan et al. [58]	1976-1980	5,915 ♂, 6,588 ♀	Use ≥ 1 time/wk	30	40	ND	ND	ND	ND	ND	ND	ND	ND
	Balluz et al. [59]	1988-1994	33,905 ♂ & ♀	Use in Last Month	35	44	ND	ND	ND	ND	ND	ND	ND	ND
	Radimer et al. [60]	1999-2000	2,260 ♂, 2,602 ♀	Use in Last month	46	57	32	38	12	13	12	14	4	16
	Kennedy et al. [61]	2007-2008	3,364 ♂ & ♀	Use in Last Month	42	54	ND	ND	ND	ND	ND	ND	ND	ND

*Abbreviations:* VM = vitamin and/or mineral; ♂ = men; ♀ = women; ND = no data; wk = week.

Among military personnel, the most common rationale for taking MVMs was to promote general health with 76% of individuals reporting this reason in one study [8]. Most MVM supplements contain at least 10 vitamins and/or minerals with a great range of dosages [62]. Systematic and narrative reviews on the effects of MVM supplements on health have indicated that there is little convincing evidence that these DSs influence the incidence of cataracts, cardiovascular disease, diabetes [63-65], or cancers of the prostate, lungs, or breast [64,66-68]. On the other hand some studies suggest MVM supplements may improve cognitive functioning [69,70], reduce infection risk in older individuals [71], reduce colon cancer risk [72] and, when used for primary prevention, reduce the risk of all-cause mortality [73], although results are not consistent [65]. The determination of the safety of vitamins and minerals differs from that of other substances like toxins or other chemicals because a certain level of vitamins and minerals are needed for good health but above or below that level, an adverse effect may occur. Thus, there is a “U-shaped” relationship between the dosage and the likelihood of adverse effects and dose–response curve will differ for different DSs [74].

Use prevalence of individual vitamins and minerals were less often reported in military studies but a few studies of Army personnel had examined Vitamin C, Vitamin E, calcium, and iron [6,12,26]. Generally, the use prevalence of Vitamin C was similar among Army men, women, and Special Forces Soldiers (15%). Vitamin E use prevalence was about the same among Army men, Army women, and elite groups (7-12%). Women appeared more likely to supplement with calcium (14%) compared to Army men (9%). Table 4 provides data on Vitamin C, Vitamin E, and calcium supplementation from the NHIS. Again, comparisons with the military data are limited for reasons cited above. Nonetheless, the NHIS and NHANES general trends were similar to the military data in that men and women were about equally likely to supplement with Vitamins C and E and women were more likely to use calcium.

Vitamin C and E are antioxidant vitamins and they have been studied in disease states like cancer, cardiovascular disease, and macular degeneration where oxidative stress mechanisms produce cellular damage [75,76]. In systematic reviews, Vitamin C and E supplementation either alone or in combination with other substances did not reduce the incidence of cardiovascular disease [64,75,77] or prostate cancer [66]. Supplemental Vitamin C may have modestly reduced the risk of breast cancer but Vitamin E did not [78]. Vitamin C and E from any source (diet or supplements) appeared to reduce the incidence of endometrial cancer [79]. Calcium supplementation with Vitamin D reduced stress fracture risk in basic training [80].

The prevalence of herbal supplement use by military personnel was small, usually 5% or less of the groups surveyed [6,17,23,29,33,39,45], although ginseng use was 14% in one Army study [6]. The nationally representative NHIS data from 2007 indicated that 17%, 12%, 12%, and 11% of the surveyed group had used Echinacea, garlic supplements, ginseng, and ginkgo biloba, respectively, in the last 30 days [81]. In athletic groups, 2% to 10% reported current use of ginseng and 4 to 7% reported current use of Echinacea [50,82,83].

Sport drinks are a general category of beverages that contain water, carbohydrates, minerals, and electrolytes, and may contain small amounts of vitamins. They are designed to be used during and after sport and exercise activities for rehydration, replacement of electrolytes lost during sweating, and energy (i.e., supply carbohydrates during activity or replenish muscle glycogen post-activity) [84,85]. Sport drinks appeared to be a very common nutritional supplement used by elite military groups, although the use prevalence range was wide (19% to 71%) in the various investigations. Sport drink use prevalence was also high among the general male and female military population (~23%) but this did not exceed the use prevalence for MVMs in these groups. The relatively high use of sport drinks among elite military groups is in consonance with studies of elite athletes showing that the use of sport drinks exceeds that of multivitamins [86-88]. Data from the NHIS indicated that 22% reported the use of sport or energy drinks ≥1 time/week but that survey did not provide data on the two beverages separately [89]. Data from NHANES indicated an almost tripling in the use of sport and energy drinks (combined) among adolescents and young adults (12 to 34 years of age) over the 1999 to 2008 period [90].

Creatine was a DS with relatively high use prevalence among elite service members (20%) and among Army men (14%), although the two studies on Army men had widely varying prevalence values [6,26]. The 2007 NHIS survey reported a 3% use prevalence in the general US population [81]. Studies on athletes have found that creatine use prevalence is highly variable and dependent on the sport. Athletes in strength and power sports (e.g., weightlifting, football, track and field) used creatine to a greater extent than those involved in endurance activities [91-94]. Research has generally shown that creatine supplementation can improve strength and performance in short-term, high intensity physical activities [95-98]. In combination with resistance training, creatine increased maximal muscle strength to a greater extent than resistance training alone [99].

In elite military groups, the use prevalence for protein supplements was about 20% but varied widely, between 12% and 47% [8,12,21,27]. The only study to report on the protein supplementation in the general male army population found a use prevalence of 13% [26], while another study reporting on combined proteins and amino acids found a use prevalence of 20% [8]. One national survey (Health and Diet Survey conducted by the US Food and Drug Administration) reported that about 1% of the total sample had used amino acid supplements in the last year [56]. The Recommended Daily Allowance for protein is 0.8 gm●kg body weight ^−1^●day^−1^[100]. However, summaries of studies indicated that the daily average intake of protein among strength-trained athletes was 2.1 gm●kg^−1^●day^−1^[101] while that among endurance athletes was 1.8 ± 0.4 gm●kg^−1^●day^−1^ for men and 1.2 ± 0.03 gm●kg^−1^●day^−1^ for women [102]. A recent consensus statement on the efficacy of protein supplementation in military personnel recommended 1.5 to 2.0 gm●kg^−1^●day^−1^ for service members involved in substantially increased metabolic demand and 1.2 to 1.5 gm●kg^−1^●day^−1^ for older service members [103]. A meta-analysis of 22 studies suggested that protein supplementation (>1.2 gm●kg^−1^●day^−1^) with resistance training resulted in modestly greater gains in fat-free mass (0.7 kg, 95% CI = 0.5-0.9 kg) and strength when compared to training without protein supplementation [104].

Three studies reported on energy drink consumption among soldiers [42] and among soldiers and marines deployed in Afghanistan [9,44]. Prevalence of energy drink usage was 25% among female soldiers [42] and 41% to 45% among male soldiers and marines [9,42,44]. There are about 500 brands of energy drinks available worldwide and about 200 are available in the US [105,106]. Literature reviews examining commonly available energy drinks found that virtually all contained caffeine, taurine, and B-Vitamins, while other common ingredients contained in most included guarana, ginseng, sugars, and carnitine. Other ingredients found in some energy drinks include ginkgo biloba, milk thistle, branched-chain amino acids, choline, chromium, green tea, hornet saliva, inositol, yerba mate, triglycerides, proline, pyruvate, royal jelly, schizandra, aloe vera, bee pollen, borage oil, and stabilized oxygen. Reviews have generally concluded that except for some relatively weak evidence for glucose and guarana, there was little evidence for a positive effect on cognitive or physical performance for any of those ingredients other than caffeine [106-108]. Caffeine has been shown to increase performance during long-term exercise, shorter-term high intensity exercise (60–180 sec), and high intensity intermittent exercise, but effects on muscle strength are equivocal. Caffeine may produce ergogenic effects through a variety of mechanisms that include increasing fat oxidation (thus sparing glucose and muscle glycogen during long-term exercise), central nervous system stimulation, a direct action on muscles, and/or a competitive inhibition with adenosine. The adenosine mechanism involves caffeine occupying adenosine sites in the central nervous system which increase catecholamine release and lipolysis. Tolerance to caffeine has often been reported and may be associated with the up-regulation of adenosine receptors [109-112].

Of interest were the studies on Marine Corps and Air Force trainees that asked about lifetime prevalence of DS use [36,39]. About 82% of the participants in these two studies were men. The lifetime prevalence of any DS use was 41% [36] and 50% [39]. These prevalences were lower than those reported by Bray et al. [19] for longer-serving Marine and AF personnel (~60%). These data suggest that a number of service members have used DSs before entering the Air Force or Marine Corps but that prevalence becomes higher once individuals spend time in these services. Military service may increase DS use because of the demands of the occupations and the belief that DSs will improve health and increase performance on occupational tasks.

In the nine investigations that examined the prevalence of DS use in elite military units [5,8,12,21,22,27,28,38],[45], all but two [22,38] reported a higher use of DSs (any use) among these elite service members compared to non-elite male military samples. This is similar to results found for elite athletes where athletes participating at higher levels of competition (Olympic, national, or international level) were more likely to use DSs than those competing as recreational athletes [49]. Elite athletes and elite service members may be similar in that they seek to gain additional physical advantages from the use of DSs.

Five studies provided information on the reasons that service members used DSs [6,8,9,19,33]. In four of the five, “general health” was listed as the reason with the highest frequency with performance enhancement listed as second most common (first in Cassler et al. study [9] of deployed marines). Thus, service members reported using DSs for the same reason as civilians, [10,11] but a second very common reason was performance enhancement which is seldom mentioned in civilian investigations. In this sense, service members are like athletes who also report performance enhancement as a high frequency reason for DS use [113-115]. Like athletes, service members’ occupational tasks require a high level of physical performance; they are unlike most athletes in that their activity may be performed in hostile locations and under adverse and austere environmental conditions.

Future studies on the prevalence of DS use should consider five major issues. First, the definition of DSs should be clearly stated on the questionnaire instructions. The legal definition provided by The Dietary Supplement Health and Education Act of 1994 [2] can serve as a standard. Second, studies should be specific about the types of DSs used by study participants. Reporting in general categories like “antioxidant”, “energy”, “herbal”, “bodybuilding”, and the like does not provide the specificity needed for comparisons across studies and the identification of specific DS use. Third, the reporting timeframe should be specific and include several periods. The most useful reporting timeframes appear to be daily, 2–6 times/week, 1 time/week and 1 time/month. Fourth, the response rate of survey should be specified and, if possible, characteristics of respondents and non-respondents should be described so that possible bias can be assessed. Finally, studies are needed that use the same experimental methods to compare DS use across all the military services over time.

There were limitations to this review. Studies differed on the reporting timeframe, questionnaire construction, and supplement definitions which made it difficult to directly compare results across all studies. In the meta-analysis, an attempt was made to control for the reporting timeframe by only examining studies asking service members about current supplement use or use ≥1 day/week. We only examined specific DSs and did not include DSs that were included in broad categories (e.g., antioxidant, ergogenics, bodybuilding). Thus, we may have underestimated DSs in some categories, although most unique studies (n = 24, 72%) did report use of “any” DS. Some questionnaires involved “checklists” of specific DSs that may have elicited better subject recall than open-ended questions asking subjects to list the DSs that they used. In most studies, the actual questionnaire structure and/or questionnaire items were not specified and the only apparent fact was that the questionnaire did or did not focus on DSs. It is possible that some service members may have been involved in one or more surveys but the number of these individuals would likely have been very small. Some values had to be estimated from graphic presentations which could have resulted in small errors. The analyses of temporal trends depended on the publication year which was likely not the year that the data were collected. Other problems common to self-reporting included the accuracy of subject recall and the possible reluctance of some individuals to report specific DSs that they used.

## Conclusion

In conclusion, this review provided a comprehensive overview of military DS use by gender and type of military service. It demonstrated that Army personnel tended to use DSs and MVM less than other service members but that regardless of service, the use of any DS and MVMs are higher among women than men. Military personnel’s use of herbal supplements is small, <5% in most investigations. Elite military men appeared to use DSs and sport drinks more than other service members.

## Abbreviations

US: United States; DS: Dietary supplement; MVM: Multivitamin/multimineral; SPE: Summary prevalence estimate; S95% CI: Summary 95% confidence Interval; SEAL: Sea, air, land (Navy Special Operations Personnel); DTIC: Defense technical information center; NHANES: National Health and Nutrition Survey; NHIS: National Health Interview Survey; 95% CI: 95% confidence interval.

## Competing interests

The authors have no competing interests.

## Authors’ contributions

JJK obtained references, compiled the data, performed the methodology quality review, performed the statistical analysis, interpreted the data, and drafted the manuscript. RS obtained references, compiled the data, performed the methodology quality review, assisted with the statistical analysis and data interpretation, and helped draft the manuscript. SSH performed the methodology quality review, assisted in data interpretation, and helped draft the manuscript. KA, EF, and HRL assisted with data interpretation and helped draft the manuscript. All authors read, commented on, and approved the final manuscript.

## Authors’ information

The views, opinions, and/or findings contained in this report are those of the authors and should not be construed as official Department of the Army position, policy, or decision, unless so designated by other official documentation. Approved for public release; distribution is unlimited.

## Pre-publication history

The pre-publication history for this paper can be accessed here:

http://www.biomedcentral.com/1472-6882/14/143/prepub

## References

[B1] Strengthening knowledge and understanding of dietary supplements [http://ods.od.nih.gov/About/DSHEA_Wording.aspx], accessed 4 February 2013

[B2] Dietary Supplement Health and Education Act of 1994 [http://www.fda.gov/RegulatoryInformation/Legislation/FederalFoodDrugandCosmeticActFDCAct/SignificantAmendmentstotheFDCAct/ucm148003.htm], accessed 11 March 2013

[B3] Saldanha LG The dietary supplement marketplace. Constantly evolving Nutr Today 2007 42 2 52 54 10.1097/01.NT.0000267126.88640.3d

[B4] Considering a post-DSHEA World Nutr Bus J 2012 17 5/6 1, 3 9

[B5] Deuster PA Sridhar A Becker WJ Coll R O’Brien KK Bathalon G Health assessment of U.S. Army Rangers Mil Med 2003 168 1 57 62 12546248

[B6] Brasfield K Dietary supplement intake in the active duty enlisted population US Army Med Dep J 2004 44 56

[B7] Boos CJ Wheble GAC Campbell MJ Tabner KC Woods DR Self-administration of exercise and dietary supplements in deployed British military personnel during operation TELIC 13 J R Army Med Corps 2010 156 1 32 36 10.1136/jramc-156-01-07 20433103

[B8] Lieberman HR Stavinoha TB McGraw SM White A Hadden LS Marriott BP Use of dietary supplements among active-duty US Army soldiers Am J Clin Nutr 2010 92 4 985 995 10.3945/ajcn.2010.29274 20668050

[B9] Cassler NM Sams R Cripe PA McGlynn AF Perry AB Banks BA Patterns and perceptions of supplement use by U.S. Marines deployed to Afghanistan Mil Med 2013 178 6 659 664 10.7205/MILMED-D-12-00440 23756073

[B10] Bailey RL Gahche JJ Miller PE Thomas PR Dwyer JT Why US adults use dietary supplements JAMA Int Med 2013 173 3 355 361 10.1001/jamainternmed.2013.2299 23381623

[B11] Dickinson A Bonci L Boyon N Franco JC Dietitians use and recommend dietary supplements: report of a survey Nutr J 2012 11 14 10.1186/1475-2891-11-14 22416673 PMC3331817

[B12] Bovill ME Tharion WJ Lieberman HR Nutrition knowledge and supplement use among elite U.S. Army soldiers Mil Med 2003 168 12 997 1000 14719624

[B13] Loney PL Chambers LW Bennett KJ Roberts JG Stratford PW Critical appraisal of health research literature: prevalence or incidence of a health problem Chronic Dis Can 2000 19 4 170 176 10029513

[B14] Carlson DE Dugan T Buchbinder J Allegetto J Schnakenberg DD Nutritional Assessment of the Ft Riley Non-Commissioned Officer Academy Dining Facility 1987 Natick MA: US Army Research Institute of Environmental Medicine Technical Report No. T14-87

[B15] Klicka MV Sherman DE King N Friedl KE Askew EW Nutritional Assessment of Cadets at the U.S. Military Academy. Part 2. Assessment of Nutritional Intake 1994 Natick MA: US Army Research Institute of Environmental Medicine Technical Report No. T94-1

[B16] Warber J McGraw S Kramer FM Lesher L Johnson W Cline A The Army Food and Nutrition Survey 1999 Natick MA: US Army Research Institute of Environmental Medicine Technical Report No. XX-99

[B17] Shanks K Prevalence of Herbal Therapy use in Active Duty Air Force Women 2001 Bethesda MD: Uniformed Services University of the Health Sciences Technical Report No. C101-88

[B18] Castillo EM Hurtado SL Shaffer RA Rock CL Brodine SK Dietary Supplement use in a Physically Active Population 2004 San Diego CA: Naval Health Research Center Technical Report

[B19] Bray RM Hourani LL Olmsted KLR Witt M Brown JM Pemberton MR Marsden ME Marriott B Scheffler S Vandermass-Peeler R Weimer B Calvin S Bradshaw M Close K Hayden D 2005 Department of Defense Survey of Health Related Behaviors Among Active Duty Personnel Research Triangle 2006 Park NC: Research Triangle Institute Technical Report No. RTI/7841/106-FR

[B20] Wells TS Webb TS Modifiable Characteristics Associated With the Training Success Among US Air Force Tactical Control Party Candidates 2010 Wright-Patterson Air Force Base OH: Air Force Research Laboratory Technical Report

[B21] Schneider K Hervig L Ensign WY Prusaczyk WK Goforth HW Use of supplements by U.S. Navy seals Med Sci Sports Exerc 1998 30 5 609475645

[B22] McGraw SM Therion WJ Lieberman HR Use of nutritional supplements by U.S. Army Rangers FASEB J 2000 14 4 A742

[B23] Corum S Greenwood MRC, Oria M Findings of Recent Surveys on Dietary Supplements Use by Military Personnel and the General Population (Appendix C) Use of Dietary Supplements by Military Personnel 2008 Washington DC: National Academy Press 384 385

[B24] French A Greenwood MRC, Oria M Findings of Recent Surveys on Dietary Supplements Use by Military Personnel and the General Population (Appendix C) Use of Dietary Supplements by Military Personnel 2008 Washington DC: National Academy Press 386 387

[B25] Marroitt BM Greenwood MRC, Oria M Findings of Recent Surveys on Dietary Supplements Use by Military Personnel and the General Population (Appendix C) Use of Dietary Supplements by Military Personnel 2008 Washington DC: National Academy Press 404 405

[B26] Lieberman H Greenwood MRC, Oria M Findings of Recent Surveys on Dietary Supplements Use by Military Personnel and the General Population (Appendix C) Use of Dietary Supplements by Military Personnel 2008 Washington DC: National Academy Press 398 399

[B27] Lieberman H Greenwood MRC, Oria M Findings of Recent Surveys on Dietary Supplements Use by Military Personnel and the General Population (Appendix C) Army Rangers Use of Dietary Supplements by Military Personnel 2008 Washington DC: National Academy Press 400 401

[B28] Lieberman H Greenwood MRC, Oria M Findings of Recent Surveys on Dietary Supplements Use by Military Personnel and the General Population (Appendix C), Special Forces Use of Dietary Supplements by Military Personne 2008 Washington DC: National Academy Press 400 401

[B29] Lieberman H Greenwood MRC, Oria M Findings of Recent Surveys on Dietary Supplements Use by Military Personnel and the General Population (Appendix C), Army War College Use of Dietary Supplements by Military Personnel 2008 Washington DC: National Academy Press 402 403

[B30] Johnson AE Greenwood MRC, Oria M Findings of Recent Surveys on Dietary Supplements Use by Military Personnel and the General Population (Appendix C) Use of Dietary Supplements by Military Personnel 2008 Washington DC: National Academy Press 414 415

[B31] Thomasos CJ Greenwood MRC, Oria M Findings of Recent Surveys on Dietary Supplements Use by Military Personnel and the General Population (Appendix C) Use of Dietary Supplements by Military Personnel 2008 Washington DC: National Academy Press 406 407

[B32] Klicka MV King N Lavin PT Askew EW Assessment of dietary intake of cadets at the US Military Academy at West Point J Am Coll Nutr 1996 15 3 273 282 10.1080/07315724.1996.10718598 8935443

[B33] Kennedy J Arsenault J Dietary supplement use in U.S. Army Special Forces Operations Candidates Mil Med 1999 164 7 495 501 10414065

[B34] Sheppard HL Raichada SM Kouri KM Stenson-Bar-Maor L Branch JD Use of creatine and other supplements by members of civilian and military health clubs: a cross-sectional survey Int J Sport Nutr Exerc Metab 2000 10 245 259 10997951 10.1123/ijsnem.10.3.245

[B35] Arsenault JE Cline AD Nutrition intakes and characteristics of normal weight, female personnel consuming foods reduced in fat or energy content Appetite 2000 34 227 233 10.1006/appe.1999.0315 10888285

[B36] Stevens MB Olsen C Ergogenic supplements and health risk behaviors J Fam Pract 2001 50 8 696 699 11509164

[B37] Smith TC Ryan MAK Smith D Reed RJ Riddle JR Gumbs GR Gray GC Complementary and alternative medicine use among US Navy and Marine Corps personnel BMC Complement Altern Med 2007 7 16 10.1186/1472-6882-7-16 17506899 PMC1884175

[B38] Johnson AE Haley CA Ward JA Hazards of dietary supplement use J Spec Oper Med 2007 7 1 30 38

[B39] Young CR Stevens MB Sports and nutritional supplement use in USMC recruits: a pilot study Mil Med 2009 174 2 158 161 10.7205/MILMED-D-00-4708 19317196

[B40] Boos CJ Simms P Morris FR Fertout M The use of exercise and dietary supplements among British soldiers in Afghanistan J R Army Med Corps 2011 157 3 229 232 10.1136/jramc-157-03-08 21977712

[B41] Carvey CE Farina EK Lieberman HR Confidence in the efficacy and safety of dietary supplements among United States active duty Army personnel BMC Complement Altern Med 2012 12 182 10.1186/1472-6882-12-182 23051046 PMC3598849

[B42] Lieberman HR Stavinoha T McGraw S White A Hadden L Marriott BP Caffeine use among active duty US Army soldiers J Acad Nutr Diet 2012 112 6 902 912 10.1016/j.jand.2012.02.001 22709816

[B43] Jacobson IG Horton JL Smith B Wells TS Boyko EJ Lieberman HR Ryan MAK Smith TC Bodybuilding, energy, and weight loss supplements are associated with deployment and physical activity in U.S. military personnel Ann Epidemiol 2012 22 5 318 330 10.1016/j.annepidem.2012.02.017 22445519

[B44] Toblin RL Clarke-Walper K Kok BC Sipos ML Thomas JL Energy drink consumption and its association with sleep problems among U.S. service members on a combat deployment--Afghanistan MMWR 2012 61 44 895 898 23134972

[B45] Kjertakov M Hristovski R Racaj M The use of dietary supplement among soldiers from the Macedonian Special Operations Regiment J Spec Oper Med 2013 13 1 19 24 23526317

[B46] Hill TM Nelson RA Consolazio CF Canham JE Nutrient Intake of the Repatriated United States Army, Navy and Marine Corps Prisoners-of-war of the Vietnam War 1978 Presidio of San Francisco: Letterman Army Institute of Research Technical Report No. 61

[B47] Jaghab S Greenwood MRC, Oria M Findings of Recent Surveys on Dietary Supplements Use by Military Personnel and the General Population (Appendix C) Use of Dietary Supplements by Military Personnel 2008 Washington DC: National Academy Press 394 395

[B48] Institutes of Medicine Use of Dietary Supplements by Military Personnel 2008 Washington DC: Institute of Medicine

[B49] Sobal J Marquart LF Vitamin/mineral supplement use among athletes: a review of the literature Int J Sports Nutr 1994 4 320 334 10.1123/ijsn.4.4.320 7874149

[B50] Huang SH Johnson K Pipe AL The use of dietary supplements and medications by Canadian athletes at the Atlanta and Sydney Olympic Games Clin J Sport Med 2006 16 1 27 33 10.1097/01.jsm.0000194766.35443.9c 16377972

[B51] Rhee KS Stubbs AC Health food users in two Texas cities J Am Diet Assoc 1976 68 542 545 1270719

[B52] Schutz HG Read M Bendel R Bhalla VS Harrill I Monagle JE Sheehan ET Standal BR Food supplement usage in seven Western states Am J Clin Nutr 1982 36 897 901 7137073 10.1093/ajcn/36.5.897

[B53] Block G Cox C Madans J Schreiber GB Melia N Vitamin supplement use, by demographic characteristics Am J Epidemiol 1988 127 4 297 309 3337084 10.1093/oxfordjournals.aje.a114805

[B54] Slesinski MJ Subar AF Kahle LL Trends in the use of vitamin and mineral supplements in the United States: the 1987 and 1992 National Health Interview Surveys J Am Diet Assoc 1995 95 8 921 923 10.1016/S0002-8223(95)00255-3 7636088

[B55] Millen AE Dodd KW Subar AF Use of vitamin, mineral nonvitamin and nonmineral supplements in the United States: the, 1992 and 2000 National Health Interview Survey results J Am Diet Assoc 1987 2004 104 942 950 10.1016/j.jada.2004.03.022 15175592

[B56] Timbo BB Ross MP McCarthy PV Lin CTJ Dietary supplements in a national survey: prevalence of use and reports of adverse events J Am Diet Assoc 2006 106 1966 1974 10.1016/j.jada.2006.09.002 17126626

[B57] Subar AF Block G Use of vitamin and mineral supplements: demographics and amount of nutrients consumed Am J Epidemiol 1990 132 6 1091 1101 2260541 10.1093/oxfordjournals.aje.a115752

[B58] Koplan JP Annest JL Layde PM Rubin GL Nutrient intake and supplementation in the United States (NHANES II) Am J Public Health 1986 76 3 287 289 10.2105/AJPH.76.3.287 3484909 PMC1646543

[B59] Balluz LS Kieszak SM Philen RM Mulinare J Vitamin and mineral supplement use in the United States Arch Fam Med 2000 9 258 262 10.1001/archfami.9.3.258 10728113

[B60] Radimer K Bindewald B Hughes J Ervin B Swanson C Picciano MF Dietary supplement use by US adults: data from the National Health and Nutrition Examination Survey,1999–2000 Am J Epidemiol 2004 160 4 339 349 10.1093/aje/kwh207 15286019

[B61] Kennedy ET Luo H Houser RF Dietary supplement use pattern of US adult population in the,2007–2008 National Health and Nutrition Survey (NHANES) Ecol Food Nutr 2013 52 76 84 10.1080/03670244.2012.706000 23282192

[B62] Huang HY Caballero B Chang S Alberg AJ Semba RD Schneyer C Wilson RF Cheng TY Prokopowicz G Barnes GJ Vassy J Bass EB Multivitamin/Multimineral Supplements and Prevention of Chronic Diseases 2007 Rockville MD: Agency for Healthcare Research and Quality (US) Technical Report No. 139

[B63] Huang HY Caballero B Chang S Alberg AJ Semba RD Schneyer C Wilson RF Cheng TY Vassy J Prokopowicz G Barnes GJ Bass EB The efficacy and safety of multivitamin and mineral supplement use to prevent cancer and chronic disease in adults: a systematic review for a National Institute of Health State-of-the-Science Conference Ann Int Med 2006 145 372 385 10.7326/0003-4819-145-5-200609050-00135 16880453

[B64] Fortmann SP Burda BU Senger CA Linn JS Whitlock EP Vitamin and mineral supplements in the primary prevention of cardiovascular disease and cancer: an updated systematic evidence review for the US Preventive Services Task Force Ann Int Med 2013 159 12 824 834 24217421 10.7326/0003-4819-159-12-201312170-00729

[B65] Sesso HD Christen WG Bubes V Smith JP MacFadyen J Schvartz M Manson JE Glynn RJ Buring JE Gaziano JM Multivitamins in the prevention of cardiovascular disease in men. The Physicians’ Health Study II Randomized Controlled Trial JAMA 2012 308 17 1751 1760 10.1001/jama.2012.14805 23117775 PMC3501249

[B66] Stratton J Godwin M The effect of supplemental vitamins and minerals on the development of prostate cancer: a systematic review and meta-analysis Fam Pract 2011 28 243 252 10.1093/fampra/cmq115 21273283

[B67] Slatore CG Littman AJ Au DH Satia JA White E Long-term use of supplemental multivitamins, Vitamin C, Vitamin E, and folate does not reduce the risk of lung cancer Am J Respir Crit Care Med 2007 177 524 530 17989343 10.1164/rccm.200709-1398OC PMC2258445

[B68] Cho E Hunter DJ Spiegelman D Albanes D Beeson WL VandenBrandt PA Colditz GA Feskanich D Folsom AR Fraser GE Freudenheim JL Giovannucci E Goldbohm RA Graham S Miller AB Rohan TE Sellers TA Virtamo J Willett WC Smith-Warner SA Intakes of Vitamins A, C, and E and folate and multivitamins and lung cancer: a pooled analysis of 8 prospective studies Int J Cancer 2006 118 970 978 10.1002/ijc.21441 16152626

[B69] Kennedy DO Haskell CF Vitamins and cognition. What is the evidence? Drugs 2011 71 15 1957 1971 10.2165/11594130-000000000-00000 21985165

[B70] Grima NA Pase MP MacPherson H Pipingas A The effects of multivitamins on cognitive performance: a systematic review and meta-analysis J Alzheimer’s Dis 2012 29 561 569 22330823 10.3233/JAD-2011-111751

[B71] Stephens AI Avenell A A systematic review of multivitamin and multimineral supplementation for infection J Hum Nutr Dietet 2006 19 179 190 10.1111/j.1365-277X.2006.00694.x 16756533

[B72] Park Y Spiegelman D Hunter DJ Albanes D Bergkvist L Buring JE Freudenheim JL Goldbohm E Hernack L Kato I Krogh V Leitzmann MF Limburg PJ Marshall JR McCullough ML Miller AB Rohan TE Schatzkin A Shore R Sieri S Stampfer MJ Virtamo J Weijenberg M Weijenberg WC Willett WC Wolk A Zhang SM Smith-Warner SA Intakes of vitamins A, C and E and use of multivitamin supplements and risk of colon cancer: a pooled analysis of prospective cohort studies Cancer Causes Control 2010 21 11 1745 1757 10.1007/s10552-010-9549-y 20820901 PMC3091388

[B73] MacPherson H Pipingas A Pase MP Multivitamin-multimineral supplementation and mortality: a meta-analysis of randomized controlled trials Am J Clin Nutr 2013 97 437 444 10.3945/ajcn.112.049304 23255568

[B74] Mulholland CA Benford DJ What is known about the safety of multivitamin-multimineral supplements for the generally healthy population? Theoretical basis for harm Am J Clin Nutr 2007 85 Suppl 318S 322S 17209218 10.1093/ajcn/85.1.318S

[B75] Nunez-Cordoba JM Martinez-Gonzales MA Antioxidant vitamins and cardiovascular disease Curr Top Med Chem 2011 11 1861 1869 10.2174/156802611796235143 21506930

[B76] Evans JR Lawrenson JG Antioxidant vitamin and mineral supplements for slowing the progression of age-related macular degeneration (review) Cochrane Database Syst Rev 2012 11:CD000254 doi:10.1002/14651858.CD000254.pub3 10.1002/14651858.CD000254.pub3 23152201

[B77] Riccioni G D’Orazio N Salvatore C Franceschelli S Pesce M Speranza L Carotenoids and Vitamins C and E in the prevention of cardiovascular disease Int J Vit Nutr Res 2012 82 1 15 26 10.1024/0300-9831/a000090 22811373

[B78] Fulan H Changxing J Baina WX Wencui Z Chunqing L Fan W Sandan L Dianjun S Tong W Da P Yashuang Z Retinol, Vitamins A, C, and E and breast cancer risk: a meta-analysis and meta-regression Cancer Causes Control 2011 22 1383 1396 10.1007/s10552-011-9811-y 21761132

[B79] Bandera EV Gifkins DM Moore DF McCullough ML Kushi LH Antioxidant vitamins and the risk of endrometrial cancer: a dose–response meta-analysis Cancer Causes Control 2009 20 5 699 711 10.1007/s10552-008-9283-x 19083131 PMC2772876

[B80] Lappe J Cullen D Haynatzki G Recker R Ahlf R Thompson K Calcium and Vitamin D supplementation decrease incidence of stress fractures in female Navy recruits J Bone Miner Res 2008 23 741 749 10.1359/jbmr.080102 18433305

[B81] Wu CH Wang CC Kennedy J Changes in herb and dietary supplement use in the US adult population: a comparison of the 2002 and 2007 National Health Surveys Clin Ther 2011 33 11 1749 1758 10.1016/j.clinthera.2011.09.024 22030445

[B82] Massad SJ Shier NW Koceja DM Ellis NT High school athletes and nutritional supplements: a study of knowledge and use Int J Sports Nutr 1995 5 3 232 245 10.1123/ijsn.5.3.232 8547941

[B83] Froiland K Koszewski W Hingst J Kopecky L Nutrition supplement use among college athletes and their sources of information Int J Sports Nutr Exerc Metabol 2004 14 104 120 10.1123/ijsnem.14.1.104 15129934

[B84] Fitness CONCOSMFCoNatCoSMa Sport drinks and energy drinks for children and adolescents: are they appropriate? Pediatrics 2011 127 1182 1189 21624882 10.1542/peds.2011-0965

[B85] Popkin BM Armstrong LE Bray GM Caballero B Frei B Willett WC A new proposed guidance system for beverage consumption in the United States Am J Clin Nutr 2006 83 529 542 16522898 10.1093/ajcn.83.3.529

[B86] Lun V Erdman KA Fung TS Reimer RA Dietary supplementation practices in Canadian high-performance athletes Int J Sports Nutr Exerc Metabol 2012 22 31 37 10.1123/ijsnem.22.1.31 22248498

[B87] Braun H Koehler K Geyer H Kleinert J Mester J Schanzer W Dietary supplement use among elite young German athletes Int J Sport Nutr Exerc Metab 2009 19 97 109 19403956 10.1123/ijsnem.19.1.97

[B88] Baylis A Cameron-Smith D Burke LM Inadvertent doping through supplement use by athletes: assessment and management of the risk in Australia Int J Sport Nutr Exerc Metab 2001 11 365 383 11591885 10.1123/ijsnem.11.3.365

[B89] Park S Onufrak S Blanck HM Sherry B Characteristics associated with consumption of sports and energy drinks among US adults: National Health Interview Survey, 2010 J Acad Nutr Diet 2013 113 112 119 10.1016/j.jand.2012.09.019 23260728 PMC4470485

[B90] Han E Powell LM Consumption patterns of sugar sweetened beverages in the United States J Acad Nutr Diet 2013 113 43 53 10.1016/j.jand.2012.09.016 23260723 PMC3662243

[B91] Greenwood M Ferris J Kreider R Greenwood L Byars A Creatine supplementation patterns and perceived effects in select Division I collegiate athletes Clin J Sport Med 2000 10 191 194 10.1097/00042752-200007000-00007 10959929

[B92] LaBotz M Smith BW Creatine supplement use in an NCAA Division I athletic program Clin J Sport Med 1999 9 167 169 10.1097/00042752-199907000-00009 10512346

[B93] Rosen O Sundgot-Borgen J Maehlum S Supplement use and nutritional habits in Norwegian elite athletes Scand J Med Sci Sports 1999 9 28 35 9974194 10.1111/j.1600-0838.1999.tb00203.x

[B94] Scofield DE Unruh S Dietary supplement use among adolescent athletes in Central Nebraska and their sources of information J Strength Cond Res 2006 20 2 452 455 16686580 10.1519/R-16984.1

[B95] Birch R Nobel D Greenhaff PL The influence of dietary creatine supplementation on performance during repeated bouts of maximal isokinetic cycling in man Eur J Appl Physiol Occ Physiol 1994 69 268 270 10.1007/BF01094800 8001541

[B96] Casey A Constantin-Teodosiu D Howell S Hultman E Greenhaff PL Creatine ingestion affects performance and muscle metabolism during maximal exercise in humans Am J Physiol Endocrinol Metabol 1996 271 E31 E37 10.1152/ajpendo.1996.271.1.E31 8760078

[B97] Balsom PD Ekblom B Soderlund K Sjodin B Hultman E Creatine supplementation and dynamic high-intensity intermittent exercise Scand J Med Sci Sports 1993 3 143 149

[B98] Gotshalk LA Volk JS Staron RS Denegar CR Hagerman FC Kraemer WJ Creatine supplementation improves muscular performance in older men Med Sci Sports Exerc 2002 34 3 537 543 10.1097/00005768-200203000-00023 11880821

[B99] Vandenberghe K Goris M VanHecke P Leemputte MV VanGerven L Hespel P Long-term creatine intake is beneficial to muscle performance during resistance exercise J Appl Physiol 1997 83 6 2055 2063 9390981 10.1152/jappl.1997.83.6.2055

[B100] Istitutes of Medicine Dietary reference intakes for energy, carbohydrates, fiber, fat, fatty acids, cholesterol, protein, and amino acids. A report of the Panel on Macronutrients, Subcommittee on Upper Levels of Nutrients and Interpretation and Uses of Dietary Reference Intake, and the Standing Committee on the Scientific Evaluation of Dietary Reference Intakes 2005 Washington DC: National Academies Press

[B101] Phillips SM Protein requirements and supplementation in strength sports Nutrition 2004 20 689 695 10.1016/j.nut.2004.04.009 15212752

[B102] Tarnopolsky M Protein requirements for endurance athletes Nutrition 2004 20 662 668 10.1016/j.nut.2004.04.008 15212749

[B103] Pasiakos SM Austin KG Lieberman HR Askew EW Efficacy and safety of protein supplements for US Armed Forces personnel: consensus statement J Nutr 2013 143 11 1811S 1814S 10.3945/jn.113.176859 24027189

[B104] Cermak NM Res PT deGroot LCPGM Saris WHM vanLoon LJC Protein supplementation augments the adaptive response of skeletal muscle to resistance-type exercise training: a meta-analysis Am J Clin Nutr 2012 96 1454 1464 10.3945/ajcn.112.037556 23134885

[B105] Reissig CJ Strain EC Griffiths RR Caffeinated energy drinks–a growing problem Drug Alcohol Depend 2009 99 1 10 10.1016/j.drugalcdep.2008.08.001 18809264 PMC2735818

[B106] Duchan E Patel ND Feucht C Energy drinks: a review of use and safety for athletes Physician Sportsmed 2010 38 2 171 179 10.3810/psm.2010.06.1796 20631477

[B107] McLellan TM Lieberman HR Do energy drinks contain active compounds other than caffeine? Nutr Rev 2012 70 12 730 744 10.1111/j.1753-4887.2012.00525.x 23206286

[B108] Seifert SM Schaechter JL Hershorin ER Lipshultz SE Health effects of energy drinks on children, adolescents, and young adults Pediatrics 2011 127 3 511 528 10.1542/peds.2009-3592 21321035 PMC3065144

[B109] Astorino TA Roberson DW Efficacy of acute caffeine ingestion for short-term high-intensity exercise performance: a systematic review J Strength Cond Res 2010 24 1 257 265 10.1519/JSC.0b013e3181c1f88a 19924012

[B110] Davis JK Green JM Caffeine and anaerobic performance. Ergogenic value and mechanisms of action Sports Med 2009 39 10 815 852 10.2165/11317770-000000000-00000 19757860

[B111] Graham TE Caffeine and exercise. Metabolism, endurance and performance Sports Med 2001 31 11 785 807 10.2165/00007256-200131110-00002 11583104

[B112] Keisler BD Armsey TD Caffeine as an ergogenic aid Curr Sports Med Rep 2006 5 215 219 10.1097/01.CSMR.0000306510.57644.a7 16822345

[B113] Krumbach CJ Ellis DR Driskell JA A report on vitamin and mineral supplement use among university athletes in a division I institution Int J Sport Nutr 1999 9 416 425 10660872 10.1123/ijsn.9.4.416

[B114] Petroczi A Naughton DP Mazanov J Holloway A Bigham J Performance enhancement with supplements: incongruence between rationale and practice J Int Soc Sports Nutr 2007 4 19 10.1186/1550-2783-4-19 17997853 PMC2214727

[B115] Kristiansen M Levy-Milne R Barr S Flint A Dietary supplement use by varsity athletes at a Canadian university Int J Sports Nutr Exerc Metabol 2005 15 195 210 10.1123/ijsnem.15.2.195 16089277

